# Supplementary material for: Causal Relationship between Aging and Anorexia Nervosa: A White-Matter-Microstructure-Mediated Mendelian Randomization Analysis
Source: Biomedicines. 2024 Aug 16;12(8):1874. doi: 10.3390/biomedicines12081874 (PMC11351342; doi:10.3390/biomedicines12081874)
Supplement: Supplementary file 1 [file biomedicines-12-01874-s001.zip › Supplementary Figure/Figure S4/Singe SNP plot of MR analysis for TL to ubm-b-1762.pdf]

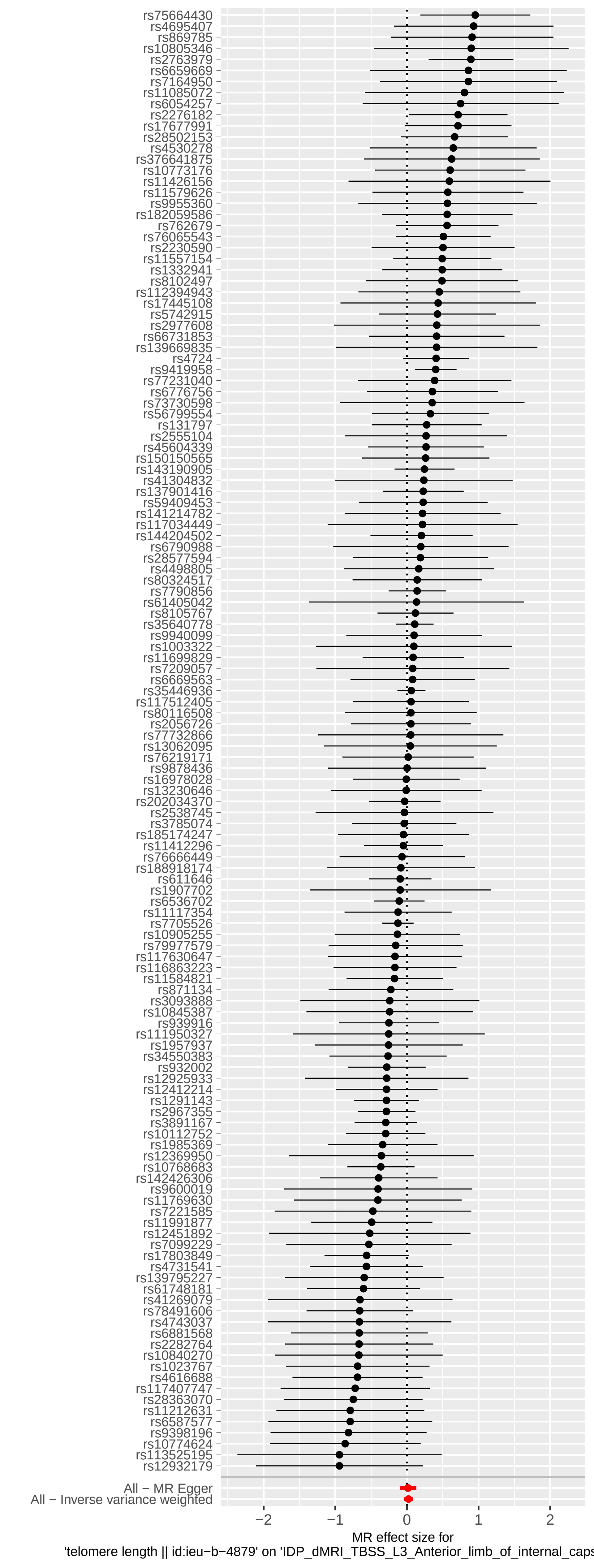
rs75664430
rs4695407
rs869785
rs10805346
rs2763979
rs6659669
rs7164950
rs11085072
rs6054257
rs2276182
rs17677991
rs28502153
rs4530278
rs376641875
rs10773176
rs11426156
rs11579626
rs9955360
rs182059586
rs762679
rs76065543
rs2230590
rs11557154
rs1332941
rs8102497
rs112394943
rs17445108
rs5742915
rs2977608
rs66731853
rs139669835
rs4724
rs9419958
rs77231040
rs6776756
rs73730598
rs56799554
rs131797
rs2555104
rs45604339
rs150150565
rs143190905
rs41304832
rs137901416
rs59409453
rs141214782
rs117034449
rs144204502
rs6790988
rs28577594
rs4498805
rs80324517
rs7790856
rs61405042
rs8105767
rs35640778
rs9940099
rs1003322
rs11699829
rs7209057
rs6669563
rs35446936
rs117512405
rs80116508
rs2056726
rs77732866
rs13062095
rs76219171
rs9878436
rs16978028
rs13230646
rs202034370
rs2538745
rs3785074
rs185174247
rs11412296
rs76666449
rs188918174
rs611646
rs1907702
rs6536702
rs11117354
rs7705526
rs10905255
rs79977579
rs117630647
rs116863223
rs11584821
rs871134
rs3093888
rs10845387
rs939916
rs111950327
rs1957937
rs34550383
rs932002
rs12925933
rs12412214
rs1291143
rs2967355
rs3891167
rs10112752
rs1985369
rs12369950
rs10768683
rs142426306
rs9600019
rs11769630
rs7221585
rs11991877
rs12451892
rs7099229
rs17803849
rs4731541
rs139795227
rs61748181
rs41269079
rs78491606
rs4743037
rs6881568
rs2282764
rs10840270
rs1023767
rs4616688
rs117407747
rs28363070
rs11212631
rs6587577
rs9398196
rs10774624
rs113525195
rs12932179
All − MR Egger
All − Inverse variance weighted
−2
−1
0
1
2
MR effect size for
'telomere length || id:ieu−b−4879' on 'IDP_dMRI_TBSS_L3_Anterior_limb_of_internal_caps
